# Effect of Hypertrophic Scar Fibroblast-Derived Exosomes on Keratinocytes of Normal Human Skin

**DOI:** 10.3390/ijms24076132

**Published:** 2023-03-24

**Authors:** Hui Song Cui, So Young Joo, Seung Yeol Lee, Yoon Soo Cho, Dong Hyun Kim, Cheong Hoon Seo

**Affiliations:** 1 Burn Institute, Department of Rehabilitation Medicine, Hangang Sacred Heart Hospital, College of Medicine, Hallym University, Seoul 07247, Republic of Korea; bioeast007@naver.com; 2 Department of Rehabilitation Medicine, Hangang Sacred Heart Hospital, College of Medicine, Hallym University, Seoul 07247, Republic of Korea; anyany98@naver.com (S.Y.J.); hamays@hanmail.net (Y.S.C.); 3 Department of Physical Medicine and Rehabilitation, College of Medicine, Soonchunhyang University Hospital, Bucheon 14158, Republic of Korea; shouletz@gmail.com; 4 Department of Rehabilitation Medicine, Kangdong Sacred Heart Hospital, College of Medicine, Hallym University, Seoul 05355, Republic of Korea; skybluever@gmail.com

**Keywords:** hypertrophic scar, fibroblast, exosome, keratinocyte, proliferation, differentiation

## Abstract

Epidermal keratinocytes are highly activated, hyper-proliferated, and abnormally differentiated in the post-burn hypertrophic scar (HTS); however, the effects of scar fibroblasts (SFs) on keratinocytes through cell–cell interaction in HTS remain unknown. Here, we investigated the effects of HTSF-derived exosomes on the proliferation and differentiation of normal human keratinocytes (NHKs) compared with normal fibroblasts (NFs) and their possible mechanism to provide a reference for clinical intervention of HTS. Fibroblasts were isolated and cultured from HTS and normal skin. Both HTSF-exosomes and NF-exosomes were extracted via a column-based method from the cell culture supernatant. NHKs were treated for 24 or 48 h with 100 μg/mL of cell-derived exosomes. The expression of proliferation markers (Ki-67 and keratin 14), activation markers (keratins 6, 16, and 17), differentiation markers (keratins 1 and 10), apoptosis factors (Bax, Bcl2, caspase 14, and ASK1), proliferation/differentiation regulators (p21 and p27), and epithelial–mesenchymal transition (EMT) markers (E-cadherin, N-cadherin, and vimentin) was investigated. Compared with NF-exosomes, HTSF-exosomes altered the molecular pattern of proliferation, activation, differentiation, and apoptosis, proliferation/differentiation regulators of NHKs, and EMT markers differently. In conclusion, our findings indicate that HTSF-derived exosomes may play a role in the epidermal pathological development of HTS.

## 1. Introduction

Hypertrophic scar (HTS) formation is the most commonly reported complication after burns, resulting in pain, pruritus, and cosmetic problems for the patient. Therapeutic options, such as moisturization, laser therapy, compression therapy, surgery, growth factors, and cell therapy, have been applied; although their effectiveness has been confirmed, a clear treatment protocol has not yet been established because the pathology of HTS remains unclear [1]. Numerous studies on the molecular basis of the pathology of HTS have indicated that several chemokines, cytokines, or growth factors play a vital role in HTS formation [2]. In particular, transforming growth factor-β1 (TGFβ1) derived from M2 type macrophages in serious or prolonged inflammation exerts a potent profibrotic effect on fibroblast proliferation and differentiation, which results in the production of excess extracellular matrix (ECM) during the proliferation phase [2]. A clinical study revealed that the levels of TGFβ in the serum of burn patients was significantly higher than those in control patients [3]. In addition, decreased levels of apoptosis in the dysregulated remodeling phase contribute to HTS formation [4]. ECM-producing myofibroblasts are a key mediator in HTS development, which is characterized by the overexpression of α-smooth muscle actin (α-SMA), type I and III collagen, fibronectin, TGFβ1 [5], interleukin-6 (IL-6), and toll-like receptor-4 (TLR-4) [6]. Moreover, levels of the anti-apoptotic factor Bcl2 increased [7], whereas those of the death receptor ligand Fas decreased in HTS fibroblasts [8].

Keratinocytes are major cellular components of the epithelium that play important roles in maintaining the skin barrier and in the wound-healing process known as re-epithelialization. Epithelialization involves the migration of keratinocytes from the wound edge to the injury site, followed by proliferation and the formation of a new epithelial layer [9]. During re-epithelialization, keratinocyte behavior is regulated by fibroblasts in a paracrine manner, although the autocrine effect of keratinocytes cannot be ignored. Fibroblast-derived keratinocyte growth factor and IL-6 strongly induce the migration and proliferation of keratinocytes [10,11,12,13]. Fibroblasts are among the primary sources of TGFβ1 [14,15], which promotes keratinocyte migration, but inhibits their proliferation in vitro [16,17]. This phenomenon has been demonstrated by an in vivo study that showed that the elimination of TGFβ signaling increased the proliferation of keratinocytes at the wound edge and led to accelerated re-epithelialization of the wound [18]. In addition, stromal cell-derived factor 1 (SDF-1) is a potent chemokine that contributes to pathologic HTS via SDF-1/CXCR4 signaling. Moreover, the level of SDF-1 was upregulated in HTS tissue and in the serum of burn patients [19]. A study demonstrated that SDF-1 secreted from fibroblasts promotes keratinocyte proliferation and epidermal thickness [20].

Exosomes are small (30–100 nm) extracellular vesicles that are released by various types of living cells [21]. Exosomes act as intercellular communicators by delivering bioactive proteins, lipids, RNAs, and DNA from donor cells to recipient cells [21]. Exosomes can regulate cellular physiological events and participate in pathological processes. Notably, exosomes play an important role in several fibrotic diseases, including liver, renal, cardiac, and skin fibrosis [22,23,24,25]. For example, exosomes released by muscle fibroblasts derived from Duchenne muscular dystrophy patients induced an increase in fibrosis markers with α-SMA, collagen, and fibronectin, and activated ERK and Akt signaling in control fibroblasts derived from age-matched subjects [25]. Recently, we found that exosomes derived from HTS fibroblasts (HTSFs) possess a potent profibrotic property, so we hypothesized that exosomes may play pathological roles in the epidermal development of HTS formation through fibroblast-keratinocyte interaction [26].

As mentioned, most studies focused on investigating the characteristics of HTSFs. However, the cell interplay with keratinocytes has rarely been studied. Moreover, fibroblast interactions with keratinocytes were explored under normal cellular physiological condition in conditioned culture medium. Fibroblasts are commonly grown in serum supplemented culture medium. In contrast, usually keratinocyte culture conditions involve a serum-free environment with supplemented growth factors, because the serum possesses the ability to induce differentiation and inhibit proliferation of keratinocytes. The different growth conditions limit studies focusing on the effects of HTSFs on keratinocytes. However, exosomes can serve as a potential solution to overcome this problem.

In the present study, we investigated the effects of HTSF-derived exosomes on the proliferation, activation, and differentiation of keratinocytes from normal skin matched with the HTS tissues. The results may provide new insights into the pathological interactions between fibroblasts and keratinocytes mediated by exosomes in the epidermal development of HTS.

## 2. Results

### 2.1. Expression of Exosome Markers

**Figure 1:**
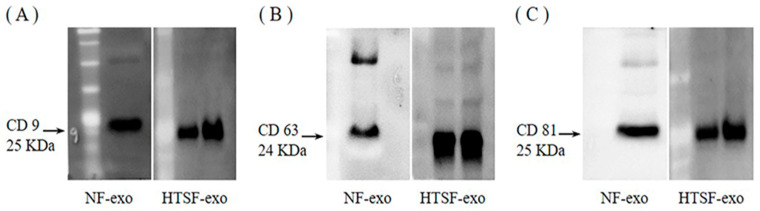
Expression of exosome surface markers. Western blot analyses of CD 9 (**A**), CD 63 (**B**), and CD 81 (**C**) expression in normal fibroblast (NF)- and hypertrophic scar fibroblast (HTSF)-exosomes. Exosomes were isolated from the serum-free culture media of fibroblasts derived from post-burn HTS. Western blot analyses were performed in triplicate.

Western blotting was performed to detect the expression of exosome markers in normal fibroblast (NF)- and HTSF-exosomes. The surface markers CD 9, CD 63, and CD 81 were expressed evidently (approximately 25, 24, and 25 kDa, respectively; Figure 1; Figure S1).

### 2.2. Effects of NF- and HTSF-Exosomes on the Proliferation of Normal Human Keratinocytes (NHKs)

**Figure 2:**
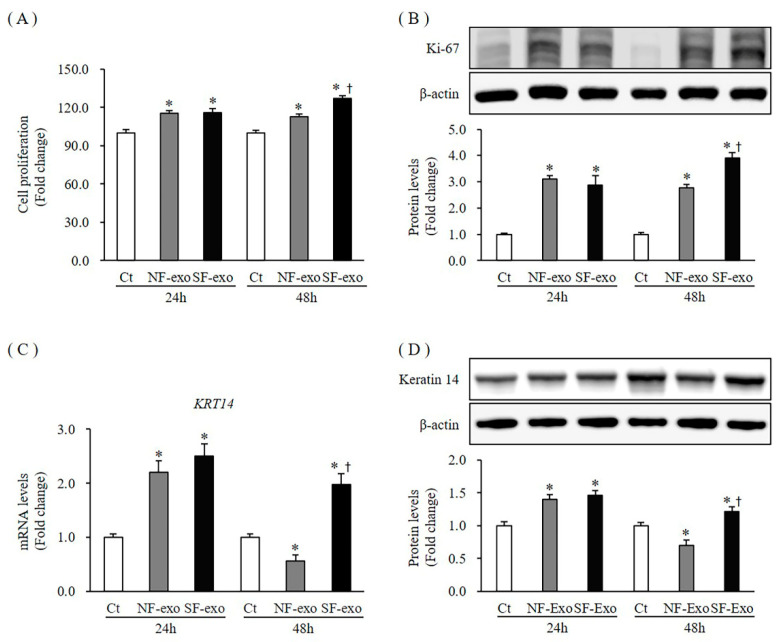
Cell proliferation and expression of proliferation markers in normal human keratinocytes (NHKs) after NF- and HTSF-exosome treatment. NHK proliferation increased significantly at 24 and 48 h after treatment with 100 μg/mL NF- and HTSF-exosomes, respectively, compared with Dulbecco phosphate-buffered saline (DPBS)-treated control cells; there was a significant difference between NF- and HTSF-exosome treatment at 48 h (**A**). For the fold change, control cells were marked by a value of 100%. The cell proliferation assay was performed in triplicate. Protein expression of Ki-67 (**B**) and the mRNA and protein expression of keratin 14 (*KRT14*; **C**,**D**) in NHKs increased significantly at 24 and 48 h after treatment with 100 μg/mL HTSF-exosomes, except for NF-exosomes at 48 h, compared with that in the control cells. * *p* < 0.05 for NF- or HTSF-exosome-treated cells versus the corresponding matched control cells. ^†^ *p* < 0.05 for HTSF-exosome-treated cells versus NF-exosome-treated cells. Data represent the mean ± standard deviation (SD); *n* = 4. Ct, DPBS-treated control cells; NHK, normal human keratinocytes; NF-exo, normal tissue-derived fibroblast exosomes; SF-exo, hypertrophic scar tissue-derived fibroblast exosomes.

To investigate the effect of NF- and HTSF-exosomes on cell growth, we first examined NHK cell proliferation. Keratinocyte proliferation increased significantly at 24 and 48 h after treatment with 100 μg/mL of NF- and HTSF-exosomes, respectively. However, at 48 h, compared with NF-exosomes, HTSF-exosomes strongly increased proliferation of NHKs compared with that of Dulbecco’s phosphate-buffered saline (DPBS)-treated control cells with an increase of 115.2% and 116% at 24 h and 112.6% and 125.1% at 48 h after treatment with 100 μg/mL NF- and HTSF-exosomes, respectively (*p* < 0.05) (Figure 2A; Figure S2).

Next, we measured protein expression of Ki-67 using Western blotting, which has been widely used as a proliferation marker [27]. The protein expression of Ki-67 was markedly increased at 24 and 48 h after treatment with 100 μg/mL of NF- and HTSF-exosomes, respectively. However, at 48 h, compared with NF-exosomes, HTSF-exosomes significantly increased Ki-67 expression (3.1- and 2.88-fold at 24 h and 2.78- and 3.9-fold at 48 h after treatment with 100 μg/mL NF- and HTSF-exosomes, respectively; *p* < 0.05) (Figure 2B; Figure S2).

Moreover, mRNA expression and protein levels of keratin 14 (*KRT14*), a proliferation marker for keratinocytes [28], increased significantly at 24 h (mRNA expression increased by 2.2- and 2.5-fold at 24 h in response to 100 μg/mL NF- and HTSF-exosomes, respectively; protein levels increased by 1.39- and 1.45-fold at 24 h in response to 100 μg/mL NF- and HTSF-exosomes, respectively; *p* < 0.05). However, the mRNA and protein expression levels of *KRT14* decreased remarkably at 48 h with NF-exosome treatment but increased at 48 h when treated with HTSF-exosomes (mRNA expression decreased by 0.56- and increased by 1.98-fold at 48 h in response to 100 μg/mL NF- and HTSF-exosomes, respectively; protein levels decreased by 0.7-fold and increased by 1.21-fold at 48 h in response to 100 μg/mL NF- and HTSF-exosomes, respectively; *p* < 0.05) (Figure 2C,D; Figure S2). These results indicate HTSF-exosomes may show a more pronounced effect in NHK proliferation than NF-exosomes.

### 2.3. Effects of NF- and HTSF- Exosomes on Expression of Activation Markers in NHKs

**Figure 3:**
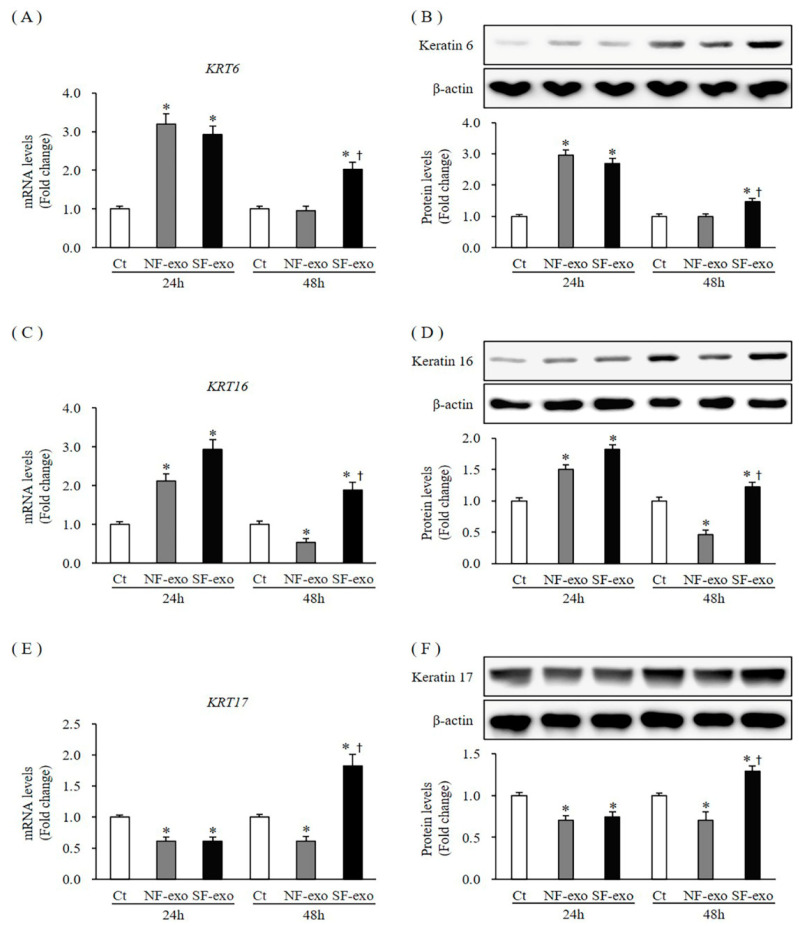
Expression of activation markers in NHKs after NF- and HTSF-exosome treatment. The mRNA and protein expression of keratin 6 (*KRT6*; **A**,**B**) in NHKs increased significantly at 24 h. However, these levels were unchanged at 48 h after treatment with 100 μg/mL NF-exosomes but increased in NHKs when treated with HTSF-exosomes compared with that in the control cells. The mRNA and protein expression of *KRT16* (**C**,**D**) increased at 24 h and decreased at 48 h after treatment with 100 μg/mL NF-exosomes but increased when treated with HTSF-exosomes. The mRNA and protein levels of *KRT17* (**E**,**F**) in NHKs decreased at 24 h after treatment with NF- and HTSF-exosomes. The mRNA and protein levels of *KRT17* decreased and increased at 48 h after treatment with NF- and HTSF-exosomes, respectively, compared with those in control cells. * *p* < 0.05 for NF- or HTSF-exosome-treated cells versus the corresponding matched control cells. ^†^ *p* < 0.05 for HTSF-exosome-treated cells versus NF-exosome-treated cells. Data represent the mean ± SD; *n* = 4. Ct, DPBS-treated control cells; NHK, normal human keratinocytes; NF, normal tissue-derived fibroblast; HTSF, hypertrophic scar tissue-derived fibroblasts.

*KRT6* and *KRT16/17* have been defined as activation markers [28]. Both the mRNA and protein expression levels of *KRT6* in NHKs increased at 24 h after treatment with NF- and HTSF-exosomes, respectively, compared with those in DPBS-treated cells (mRNA expression increased by 3.2- and 2.93-fold at 24 h in response to 100 μg/mL NF- and HTSF-exosomes, respectively; protein levels increased by 2.96- and 2.68-fold at 24 h in response to 100 μg/mL NF- and HTSF-exosomes, respectively; *p* < 0.05). However, the mRNA and protein expression levels of *KRT6* were unchanged at 48 h after treatment with NF-exosomes but increased after treatment with HTSF-exosomes (mRNA expression was 0.95- and 2.02-fold and protein levels were 1.00- and 1.47-fold at 48 h in response to 100 μg/mL NF- and HTSF-exosomes, respectively; *p* < 0.05) (Figure 3A,B; Figure S3). The mRNA expression and protein levels of *KRT16* increased at 24 h (mRNA expression increased by 2.11- and 2.93-fold and protein levels increased by 1.5- and 1.81-fold in response to 100 μg/mL NF- and HTSF-exosomes, respectively; *p* > 0.05) (Figure 3C,D; Figure S3). However, these levels decreased significantly at 48 h when treated with NF-exosomes but increased after HTSF-exosome treatment (mRNA expression was 0.53- and 1.89-fold and protein levels were 0.45- and 1.22-fold in response to 100 μg/mL NF- and HTSF-exosomes, respectively; *p* < 0.05) (Figure 3C,D; Figure S3). Moreover, the mRNA expression and protein levels of *KRT17*, a keratin 16 partner, decreased at 24 h (mRNA expression decreased by 0.62- and 0.61-fold and protein levels decreased by 0.70- and 0.75-fold in response to 100 μg/mL NF- and HTSF-exosomes, respectively; *p* < 0.05), whereas these levels at 48 h were similar to those of *KRT16* (the mRNA expression was 0.61- and 1.86-fold and protein levels were 0.70- and 1.29-fold in response to 100 μg/mL NF- and HTSF-exosomes, respectively, *p* > 0.05) (Figure 3E,F; Figure S3). These results suggest NHK activation occurs early (at 24 h) when treated with NF-exosomes; however, in response to HTSF-exosome treatment, the activation of NHKs increased early and continued for longer (48 h).

### 2.4. Effects of NF- and HTSF-Exosomes on the Expression of Differentiation Markers in NHKs

**Figure 4:**
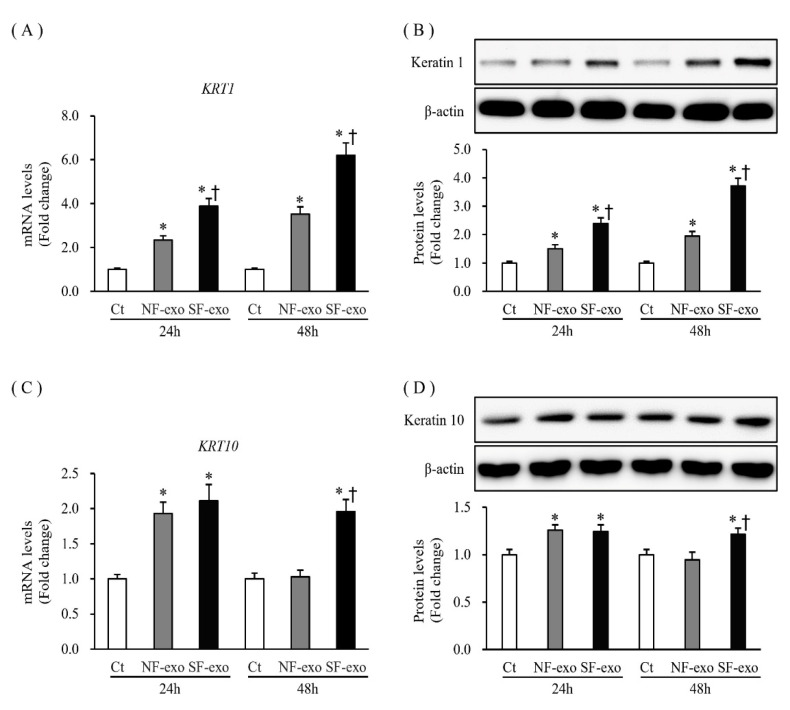
Expression of differentiation markers in NHKs after NF- and HTSF-exosome treatment. The mRNA and protein expression of *KRT1* (**A**,**B**) in NHKs increased significantly at 24 and 48 h after treatment with 100 μg/mL NF- or HTSF-exosomes, compared with that in the control cells; mRNA and protein expression of *KRT1* significantly increased after treatment with HTSF-exosomes compared with NF-exosomes. The mRNA and protein expression of *KRT10* (**C**,**D**) in NHKs increased and was unchanged at 24 h and 48 h after treatment with 100 μg/mL NF-exosomes, respectively; however, mRNA and protein expression of *KRT10* increased at 24 h and 48 h after treatment with 100 μg/mL HTSF-exosomes, compared with that in control cells. * *p* < 0.05 for NF- or HTSF-exosome-treated cells versus the corresponding matched control cells. ^†^ *p* < 0.05 for HTSF-exosome-treated cells versus NF-exosome-treated cells. Data represent the mean ± SD; n = 4. Ct, DPBS-treated control cells; NHK, normal human keratinocytes; NF, normal tissue-derived fibroblasts; HTSF, hypertrophic scar tissue-derived fibroblasts.

*KRT1* and *KRT10* are early differentiation markers of keratinocytes [28]. The mRNA and protein expression of *KRT1* increased significantly at 24 and 48 h after treatment with NF- and HTSF-exosomes, respectively, compared with that in DPBS-treated control cells. Moreover, compared with NF-exosome treatment, mRNA and protein expression of *KRT1* significantly increased at 48 h after HTSF-exosome treatment (compared with that in the DPBS-treated control cells; mRNA expression increased by 2.33- and 3.89-fold at 24 h and 3.52- and 6.21-fold at 48 h after treatment with 100 μg/mL NF- and HTSF-exosomes, respectively; protein levels increased by 1.50- and 2.39-fold at 24 h and 1.94- and 3.71-fold at 48 h in response to 100 μg/mL NF- and HTSF-exosomes, respectively; *p* < 0.05) (Figure 4A,B; Figure S4). The mRNA and protein expression of *KRT10* increased at 24 h and was unchanged at 48 h after treatment with NF-exosomes, compared with that in the DPBS-treated cells, but significantly increased at 24 h and 48 h after treatment with HTSF-exosomes, compared with that in the DPBS-treated cells (mRNA expression was 1.93- and 2.11-fold at 24 h and 1.03- and 1.96-fold at 48 h; protein levels were 1.25- and 1.24-fold at 24 h and 0.94- and 1.21-fold at 48 h in response to 100 μg/mL NF- and HTSF-exosomes, respectively; *p* < 0.05) (Figure 4C,D; Figure S4). These results suggest that HTSF-exosomes substantially induced the differentiation of NHKs more than NF-exosomes.

### 2.5. Effects of NF- and HTSF-Exosomes on the Expression of Apoptosis-Related Factors Related to Proliferation and Differentiation in NHKs

**Figure 5:**
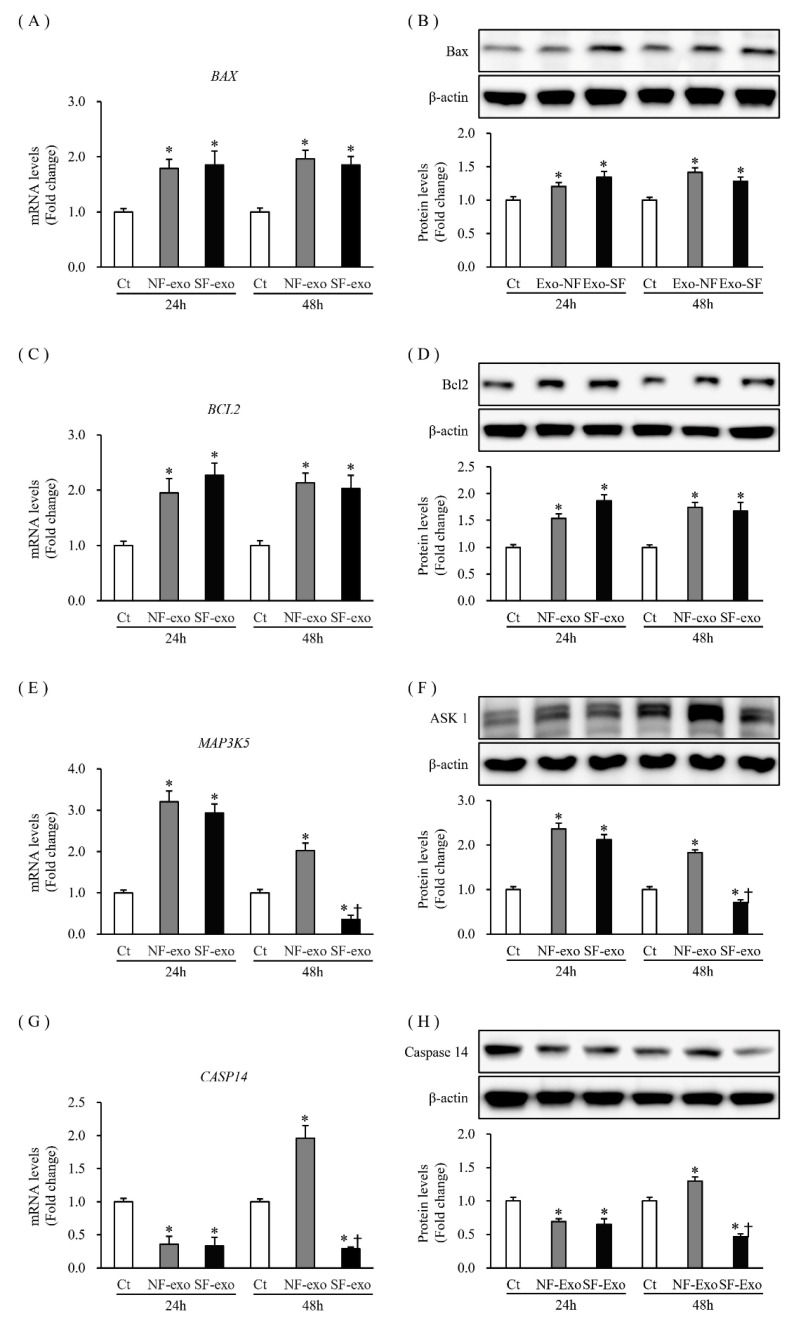
Expression of apoptosis-related factors in NHKs after NF- or HTSF-exosome treatment. The mRNA and protein expression of Bax (*BAX*) (**A**,**B**) and Bcl2 (**C**,**D**) in NHKs increased significantly at 24 and 48 h after treatment with 100 μg/mL NF- or HTSF-exosomes, compared with that in the control cells. The mRNA and protein expression of ASK1 (*MAP3K5*) (**E**,**F**) in NHKs increased significantly at 24 h after treatment with 100 μg/mL NF- or HTSF-exosomes, compared with that in the control cells. The mRNA and protein expression of *MAP3K5* increased and decreased at 48 h after treatment with NF- and HTSF-exosomes, respectively (**E**,**F**). The mRNA and protein expression of caspase 14 (*CASP14*) (**G**,**H**) in NHKs decreased significantly at 24 h after treatment with 100 μg/mL NF- or HTSF-exosomes, respectively, compared with that in the control cells. The mRNA and protein expression of *CASP14* increased and decreased at 48 h after treatment with NF- and HTSF-exosomes, respectively (**G**,**H**). * *p* < 0.05 for NF- or HTSF-exosome-treated cells versus the corresponding matched control cells. ^†^ *p* < 0.05 for HTSF-exosome-treated cells versus NF-exosome-treated cells. Data represent the mean ± SD; n = 4. Ct, DPBS-treated control cells; NHK, normal human keratinocytes; NF, normal tissue-derived fibroblasts; HTSF, hypertrophic scar tissue-derived fibroblasts.

Keratinocyte apoptosis is closely involved in epidermal development; the expression of apoptotic factors also reflects the state of cellular proliferation and differentiation, including Bax, Bcl2, apoptosis signal-regulating kinase 1 (ASK1), and Notch 1 [28]. The mRNA expression and protein levels of the pro-apoptotic factor, Bax (*BAX*), increased significantly at 24 and 48 h after NF- and HTSF-exosome treatment, respectively (mRNA levels increased by 1.79- and 1.85-fold at 24 h and 1.96- and 1.85-fold at 48 h after treatment with 100 μg/mL NF- and HTSF-exosomes, respectively; protein levels increased by 1.2- and 1.34-fold at 24 h and 1.41- and 1.28-fold at 48 h after treatment with 100 μg/mL NF- and HTSF-exosomes, respectively; *p* < 0.05) (Figure 5A,B; Figure S5). The expression of the anti-apoptotic factor, Bcl2, at the mRNA and protein levels increased significantly at 24 and 48 h (mRNA expression increased by 1.95- and 2.27-fold at 24 h and 2.13- and 2.03-fold at 48 h after treatment with 100 μg/mL NF- and HTSF-exosomes, respectively; protein levels increased by 1.53- and 1.86-fold at 24 h and 1.41- and 1.28-fold at 48 h after treatment with 100 μg/mL NF- and HTSF-exosomes, respectively; *p* < 0.05) (Figure 5C,D; Figure S5). The mRNA and protein expression of ASK1 (*MAP3K5*) increased significantly at 24 h (mRNA levels increased by 3.2- and 2.93-fold after treatment with 100 μg/mL NF- and HTSF-exosomes, respectively; protein levels increased by 2.36- and 2.12-fold after treatment with 100 μg/mL NF- and HTSF-exosomes, respectively; *p* < 0.05) (Figure 5E,F; Figure S5). The mRNA and protein expression of ASK1 (*MAP3K5*) increased at 48 h after treatment with NF-exosomes, but decreased with HTSF-exosome treatment at 48 h (mRNA levels were 2.02- and 0.36-fold and protein levels were 1.83- and 0.71-fold in response to 100 μg/mL NF- and HTSF-exosomes, respectively) (Figure 5E,F; Figure S5). The mRNA and protein expression of caspase 14 (*CASP 14*) decreased significantly at 24 h after treatment with NF- or HTSF-exosomes, compared with that in the control cells (mRNA expression decreased by 0.36- and 0.33-fold after treatment with 100 μg/mL NF- and HTSF-exosomes, respectively; protein expression decreased by 0.69- and 0.65-fold after treatment with 100 μg/mL NF- and HTSF-exosomes, respectively; *p* < 0.05) (Figure 5G,H; Figure S5). Furthermore, mRNA and protein expression of *CASP 14* at 48 h increased after NF-exosome treatment, but decreased with HTSF-exosome treatment (mRNA levels were 1.96- and 0.29-fold after treatment with 100 μg/mL NF- and HTSF-exosomes, respectively; protein levels were 1.29- and 0.46-fold in response to 100 μg/mL NF- and HTSF-exosomes, respectively; *p* < 0.05) (Figure 5G,H; Figure S5). These results further suggest that NF- or HTSF-exosome treatment regulates the expression of apoptosis-related factors in NHKs.

### 2.6. Effects of NF- and HTSF-Exosomes on the Expression of Cyclin-Dependent Kinase Inhibitors and Their Regulators in NHKs

**Figure 6:**
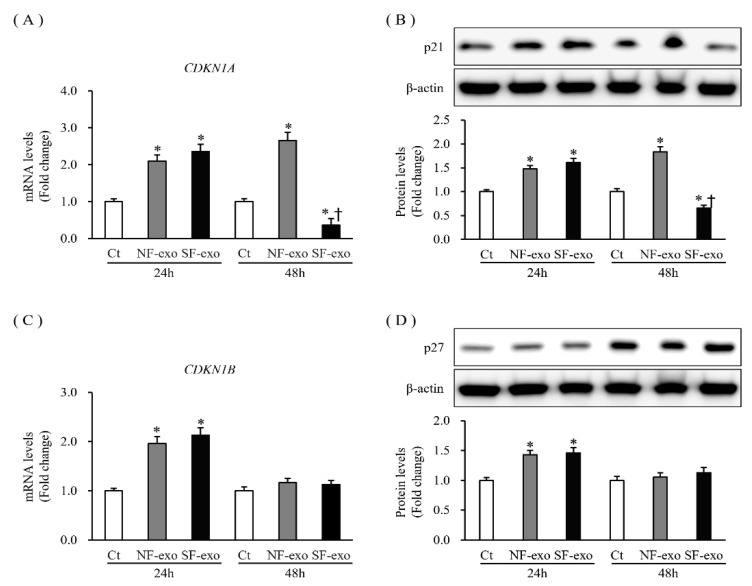
Expression of cyclin-dependent kinase inhibitors in NHKs after NF- or HTSF-exosome treatment. The mRNA and protein expression of p21 (*CDKN1A*) (**A**,**B**) in NHKs increased significantly at 24 and 48 h after treatment with 100 μg/mL NF- or HTSF-exosome treatment but decreased with HTSF-exosome treatment at 48 h compared with that in the control cells. The mRNA and protein expression of p27 (*CDKN1B*) (**C**,**D**) in NHKs increased significantly at 24 h and was unchanged at 48 h after treatment with 100 μg/mL NF- or HTSF-exosomes, respectively, compared with that in the control cells. * *p* < 0.05 for NF- or HTSF-exosome-treated cells versus the corresponding matched control cells. ^†^ *p* < 0.05 for HTSF-exosome-treated cells versus NF-exosome-treated cells. Data represented with mean ± SD; n = 4. Ct, DPBS-treated control cells; NHK, normal human keratinocytes; NF, normal tissue-derived fibroblasts; HTSF, hypertrophic scar tissue-derived fibroblasts.

p21 and p27 are cyclin-dependent kinase inhibitors that regulate the cell cycle and are associated with the proliferation and differentiation of keratinocyte phenotypes [29]. The p21 (*CNKN1A*) mRNA and protein expression levels increased significantly at 24 and 48 h (mRNA expression increased by 1.75- and 1.82-fold after 24 h and 1.97- and 2.28-fold after 48 h at 100 μg/mL, respectively; protein levels increased by 1.23- and 1.27-fold after 24 h and 1.66- and 1.67-fold after 48 h at 100 μg/mL, respectively; *p* < 0.05) (Figure 6A,B; Figure S6). However, p27 (*CDKN1B*) expression remained unchanged at 24 h and increased significantly at 48 h (at 24 h, mRNA expression was 1.09- and 1.10-fold and protein levels were 1.09- and 1.10-fold higher at 100 μg/mL, respectively, *p* > 0.05; at 48 h, mRNA expression was 1.98- and 3.17-fold and protein levels were 1.57- and 1.82-fold higher at 100 μg/mL, respectively; *p* < 0.05) (Figure 6C,D; Figure S6). These results indicate that NF-exosome treatment and, especially. HTSF-exosome treatment regulated the expression of p21 and p27 in NHKs.

**Figure 7:**
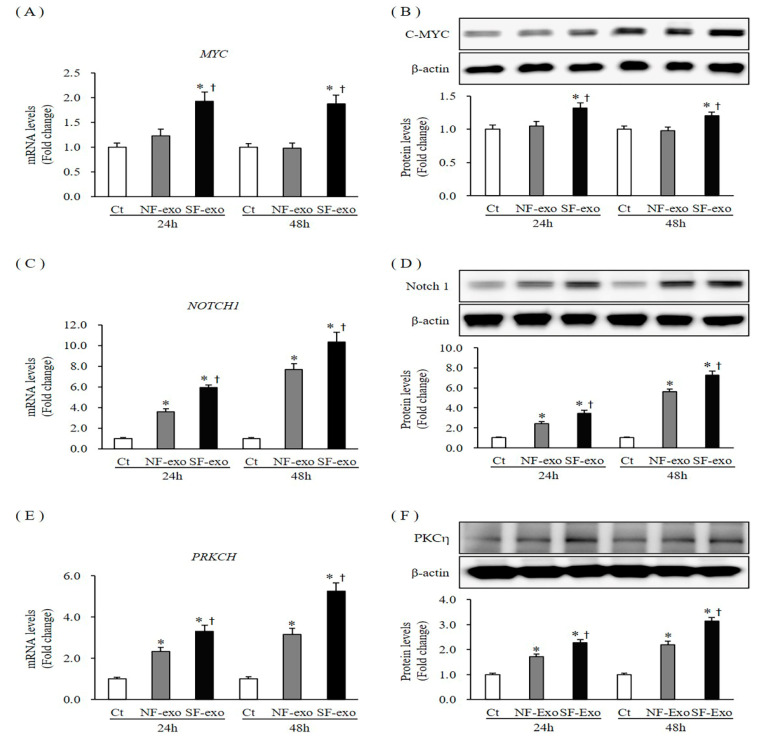
Expression of the regulators of the cyclin-dependent kinase inhibitor in NHKs after NF-and HTSF-exosome treatment. The mRNA and protein expression of C-MYC (**A**,**B**) in NHKs increased significantly at 24 and 48 h after treatment with 100 μg/mL HTSF-exosomes but remained unchanged after treatment with NF-exosomes, compared with that in the control cells. The mRNA and protein expression of Notch1 (**C**,**D**) and PKCη (**E**,**F**) increased significantly at 24 and 48 h after treatment with 100 μg/mL NF- and HTSF-exosomes, compared with that in the control cells. The mRNA and protein expression of Notch1 and PKCη increased to a greater degree with HTSF-exosome treatment than with NF-exosome treatment. * *p* < 0.05 for NF- or HTSF- exosome-treated cells versus the corresponding matched control cells. ^†^ *p* < 0.05 for HTSF-exosome-treated cells versus NF-exosome-treated cells. Data represent the mean ± SD; n = 4. Ct, DPBS-treated control cells; NHK, normal human keratinocytes; NF, normal tissue-derived fibroblasts; HTSF, hypertrophic scar tissue-derived fibroblasts.

The expression of p21 and p27 is tightly regulated by several factors, such as C-MYC, Notch1, and PKCη [30,31,32]. The C-MYC (*MYC*) mRNA and protein levels remained unchanged at 24 h and 48 h after treatment with 100 μg/mL NF-exosomes, compared with DPBS-treated control cells (mRNA expression was 1.13- and 0.98- fold at 24 and 48 h, protein levels were 1.04- and 0.97-fold and 24 and 48 h, respectively). However, *MYC* mRNA and protein levels significantly increased at 24 h and 48 h after treatment with 100 μg/mL HTSF-exosomes, compared with DPBS-treated control cells and NF-exosome treated cells (mRNA expression was 1.93- and 1.88- fold at 24 and 48 h and protein levels were 1.32- and 1.23-fold and 24 and 48 h, respectively, *p* < 0.05) (Figure 7A,B; Figure S7). Compared with NF-exosomes, mRNA and protein expression of *MYC* more significantly increased with HTSF-exosome treatment at 24 and 48 h (Figure 7A,B; Figure S7). The mRNA and protein level expression of Notch1 (*NOTCH1*) increased significantly at 24 and 48 h after treatment with 100 μg/mL NF- or HTSF-exosomes, respectively, compared with DPBS treatment (mRNA expression was 3.6- and 5.9-fold at 24 h and 7.69- and 10.32-fold at 48 h, respectively; protein levels were 2.39- and 3.45-fold at 24 h and 5.6- and 7.25-fold at 48 h, respectively; *p* < 0.05) (Figure 7C,D; Figure S7). Furthermore, compared with NF-exosomes, mRNA and protein level expression of *NOTCH1* was more significantly increased by HTSF-exosome treatment at 24 and 48 h (*p* < 0.05) (Figure 7C,D; Figure S7). The mRNA and protein expression of PKCη (*PRKCH*) increased consistently from 24 to 48 h after treatment with 100 μg/mL NF- and HTSF-exosomes compared with control, respectively (mRNA expression was 2.33- and 3.31-fold at 24 h and 3.16- and 5.26-fold at 48 h, respectively; protein levels were 1.7- and 2.16-fold at 24 h and 2.19- and 3.12-fold at 48 h, respectively; *p* < 0.05) (Figure 7E,F; Figure S7). Compared with NF-exosomes, mRNA and protein expression of *PRKCH* more significantly increased with HTSF-exosome treatment at 24 and 48 h (Figure 7E,F; Figure S7). These results suggest that NF-exosome treatment and, especially, HTSF-exosome treatment upregulated the expression of C-MYC, Notch1, and PKCη in NHKs.

### 2.7. Effects of NF- and HTSF-Exosomes on the Epithelial–Mesenchymal Transition (EMT) in NHKs

**Figure 8:**
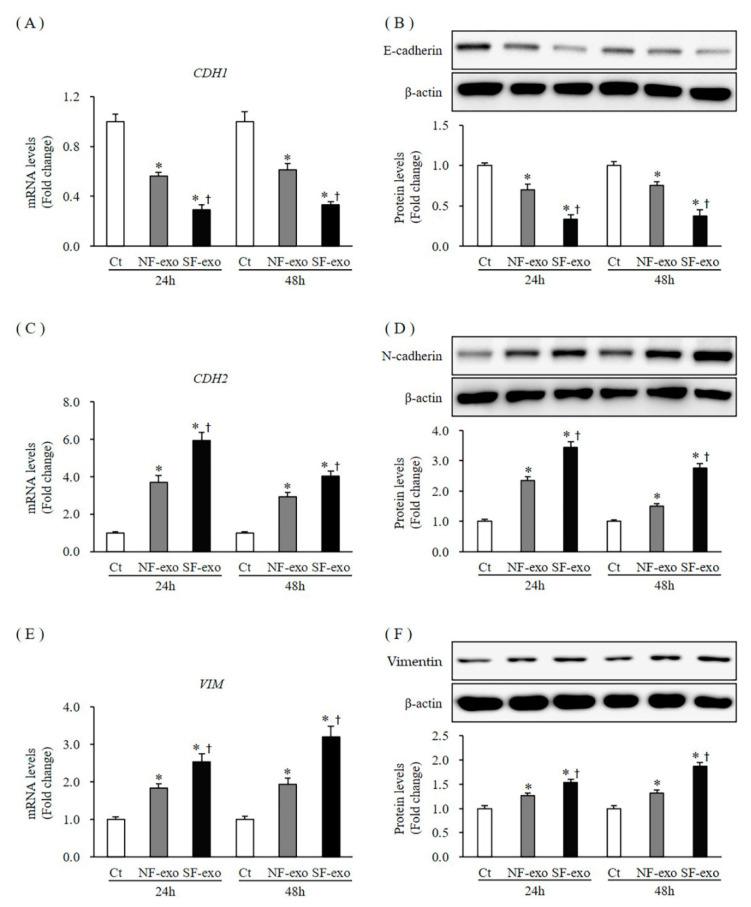
Expression of EMT markers in NHKs after NF- and HTSF-exosome treatment. The mRNA and protein expression of E-cadherin (**A**,**B**) in NHKs decreased significantly at 24 and 48 h after treatment with 100 μg/mL NF-exosome, compared with that in the control cells. They further decreased with HTSF-exosome treatment compared with NF-exosome treatment at 24 and 48 h (**A**,**B**). The mRNA and protein expression of N-cadherin (**C**,**D**) and vimentin (**E**,**F**) increased significantly at 24 and 48 h after treatment with 100 μg/mL NF-exosomes, compared with that in the control cells. They were further increased with HTSF-exosome treatment compared with NF-exosomes at 24 and 48 h (**C**–**F**). * *p* < 0.05 for NF- or HTSF-exosome-treated cells versus the corresponding matched control cells. ^†^ *p* < 0.05 for HTSF-exosome-treated cells versus NF-exosome-treated cells. Data represent the mean ± SD; n = 4. EMT, epithelial–mesenchymal transition; Ct, DPBS-treated control cells; NHK, normal human keratinocytes; NF, normal tissue-derived fibroblasts; HTSF, hypertrophic scar tissue-derived fibroblasts.

**Figure 9:**
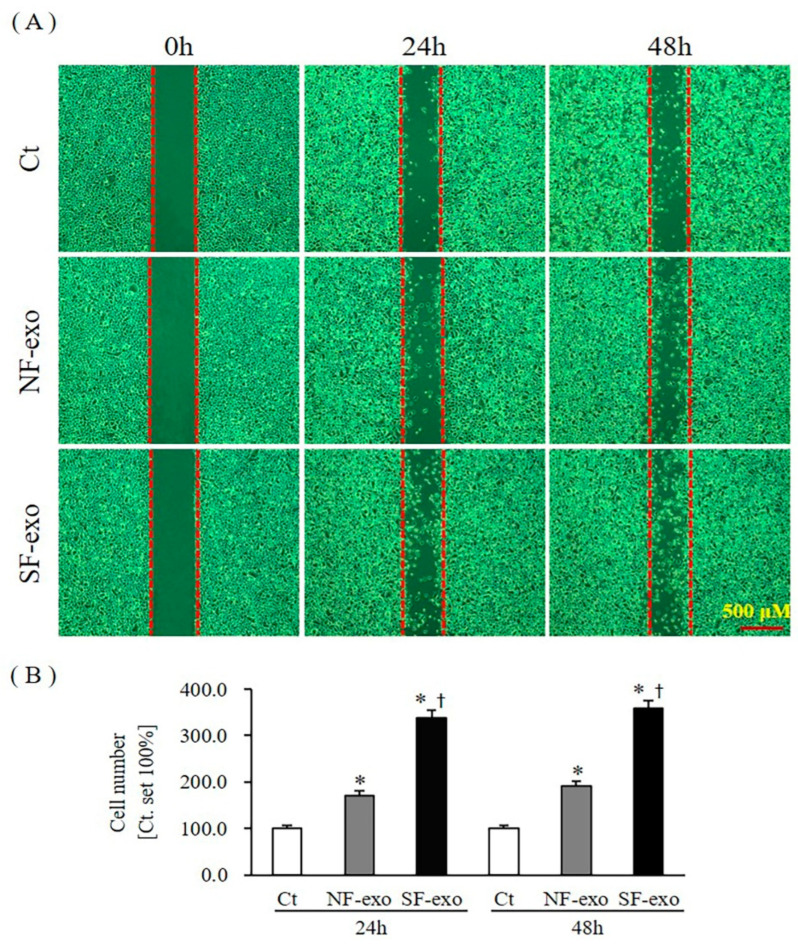
HTSF-exosome treatment dominantly increased the migration of NHKs. NHKs were treated with 100 μg/mL of NF- or HTSF-exosomes, and the control cells were treated with DPBS. After 24 and 48 h, migration of the cells was photographed (scale bar: 500 μm) (**A**). The quantitative analysis of cell migration was expressed as a percentage relative to the control cells (**B**). Ct, DPBS-treated control cells. * *p* < 0.05 for NF- or HTSF-exosome-treated cells versus the corresponding matched control cells. ^†^ *p* < 0.05 for HTSF-exosome-treated cells versus NF-exosome-treated cells. Data represent the mean ± SD; n = 4. EMT, epithelial–mesenchymal transition; Ct, DPBS-treated control cells; NHK, normal human keratinocytes; NF, normal tissue-derived fibroblasts; HTSF, hypertrophic scar tissue-derived fibroblasts.

E-cadherin, N-cadherin, vimentin, and cell migration are well-known EMT markers [33]. The mRNA and protein levels of E-cadherin (CDH1) decreased significantly at 24 and 48 h after 100 μg/mL NF-exosome treatment, compared with DPBS treatment, respectively (mRNA levels were 0.56- and 0.61-fold and protein levels were 0.70- and 0.75- fold at 24 and 48 h, respectively; *p* < 0.05) (Figure 8A,B; Figure S8). Moreover, the mRNA and protein levels of CDH1 further decreased with 100 μg/mL HTSF-exosome treatment at 24 and 48 h, respectively (mRNA levels were 0.29- and 0.33-fold and protein levels were 0.33- and 0.37- fold at 24 and 48 h, respectively; *p* < 0.05) (Figure 8A,B; Figure S8). The mRNA and protein levels of N-cadherin (CDH2) increased significantly at 24 and 48 h after 100 μg/mL NF-exosome treatment, compared with DPBS treatment, respectively (mRNA levels were 3.71- and 2.93-fold and protein levels were 2.32- and 1.53- fold at 24 and 48 h, respectively, *p* < 0.05) (Figure 8C,D; Figure S8). Moreover, the mRNA and protein levels of CDH2 further increased with 100 μg/mL HTSF-exosome treatment at 24 and 48 h, respectively (mRNA levels were 5.91- and 4.13-fold and protein levels were 3.42- and 2.83- fold at 24 and 48 h, respectively; *p* < 0.05) (Figure 8C,D; Figure S8). The mRNA and protein expression of vimentin (VIM) increased significantly at 24 and 48 h after 100 μg/mL NF-exosome treatment, compared with DPBS treatment, respectively (mRNA levels were 1.83- and 1.93-fold and protein levels were 1.25- and 1.31- fold at 24 and 48 h, respectively, *p* < 0.05) (Figure 8E,F; Figure S8). Moreover, the mRNA and protein expression of VIM further increased with 100 μg/mL HTSF-exosome treatment at 24 and 48 h, respectively (mRNA levels were 2.53- and 3.21-fold and protein levels were 1.54- and 1.91- fold at 24 and 48 h, respectively; *p* < 0.05) (Figure 8E,F; Figure S8). Cell migration also increased significantly at 24 and 48 h after 100 μg/mL NF-exosome treatment, compared with DPBS treatment, respectively (an increase of 170.3% and 191.6% at 24 h and 48 h, respectively; *p* < 0.05) (Figure 9A,B). Moreover, migration of NHKs further increased with 100 μg/mL HTSF-exosome treatment at 24 and 48 h, respectively (an increase of 338.6% and 358.7% at 24 and 48 h, respectively; *p* < 0.05) (Figure 9A,B). These results suggest that HTSF-exosome treatment strongly induced EMT, compared with NF-exosomes in NHKs.

## 3. Discussion

Post-burn HTS are conventionally attributed to the pathological development of dermis after injury. Wide-ranging studies have focused on the performance of fibroblasts but have not considered the role of epidermal keratinocytes. Nevertheless, fibroblast proliferation and dermal thickness increased upon the addition of HTS keratinocytes, compared with normal skin keratinocytes, in a tissue-engineered model [34]. The expression of IL-1α, which inhibits collagen metabolism, decreased and the expression of platelet-derived growth factor (PDGF), which upregulates dermal matrix, increased in the epidermis of HTS compared with normal scar tissues at 12 months post-surgery [35]. The epidermal expression of IL-1α and PDGF in HTS can predict whether the scar will maintain hypertrophic nature. These results suggest epidermis participation in post-burn HTS development. However, few studies have focused on the pathological characteristics of keratinocytes as the main components of epithelium. A few studies using immunohistochemistry staining showed that KRT6 and KRT16 (activation markers) were highly expressed in the interfollicular epidermis of hypertrophic scars than in the matched control sample from the same patients, while the expression of KRT1 and KRT10 (differentiation markers) was not reduced. However, the expression of another differentiation marker, filaggrin, increased [36]. In addition, focus on biological processes showed that keratinocytes in HTs expressed high levels of KRT5 (proliferation marker), KRT16 (activation marker), and filaggrin (differentiation marker) at one month after a burn. KRT16 and KRT17 (activation marker) were still upregulated at four months in HTS [37]. Moreover, both filaggrin and involucrin (differentiation marker) were at abnormal levels in HTS tissues [38]. Recently, we also reported that levels of KRT1, KRT6, KRT17, and involucrin increased in keratinocytes derived from HTS compared with their levels in matched NHKs [39]. Conventionally, however, the filaggrin expression is reduced or absent in hyperproliferative diseases, such as psoriasis and atopic dermatitis [40,41]. These results suggest that the epidermal keratinocytes in HTS display an activated phenotype and have an alternative differentiation program. Although epidermal activation has been observed, it is difficult to conclude whether it is only a consequence of HTS formation or a causal relationship with the pathogenesis.

Several studies have documented the roles of exosomes as a mechanism of cell-to-cell communication, especially as biomarkers and as a therapeutic target in fibrosis. Furthermore, exosomes derived from primary human lung fibroblasts (PHLFs) and the bronchoalveolar lavage fluid of patients with idiopathic pulmonary fibrosis increased the proliferation of PHLFs via Wnt family member 5A (WNT-5A) signaling [42]; exosomes released by cardiac myofibroblasts induced cardiac endothelial cell dysfunction by reduction of vascular endothelial growth factor (VEGF)-A, hypoxia-inducible factor-1α, CD31, and angiopoietin-1 gene expression, cell migration, and tube formation [24]; and exosomes derived from injured tubular epithelial cells can be transferred to recipient cells, leading to their proliferation and differentiation along with increasing the expression of α-SMA, F-actin, and type I collagen [23]. These studies indicate that the exosomes derived from pathological conditions differ from those in the physiological state and contribute to the pathology. In addition, exosomes derived from different subtypes of stem cells exhibit therapeutic potential and are expected to be an alternative means of developing strategies for curing various diseases [43]. The profibrotic potency of HTSF-exosomes was demonstrated by promotion of human NF proliferation and differentiation by induction of Smad-dependent and non-Smad-dependent signaling [26]. HTSF-exosomes contain large amounts of miRNA related to fibrosis, compared with those in NFs [26]. In addition, exosomes are reported to be involved in the development of organ fibrosis [44]. Therefore, the HTSF-exosomes can affect epidermal keratinocyte phenotypes. Notably, in the present study, the expression of KRT6, 16, and 17 was increased in NHKs after exposure to HTSF-exosomes. These events are also found in other hyperproliferative diseases, such as psoriasis [45].

In the dermal tissue, apoptosis controls the myofibroblast density to resemble ECM deposition. Therefore, the reduced apoptosis level is an important factor in HTS formation [4]. In epidermal cells, apoptosis maintains their thickness by balancing cell proliferation and differentiation through “an apoptotic gene program” [46]. For example, proliferating basal keratinocytes highly express Bcl-2, which is absent in suprabasal keratinocytes [47]; a gradient of Bax expression increases from lower to upper layers to promote cell death [48] and Bax expression is stronger in suprabasal keratinocytes than in basal keratinocytes [49]. In addition, the induction of ASK1 not only results in morphological changes in keratinocytes without any symptoms of the apoptotic phenotype, as evaluated by TUNEL staining, but also strongly expresses differentiation markers involucrin and transglutaminase-1 [50]. Caspase-14 is expressed only in the skin, unlike other known caspases that are expressed ubiquitously [51]. The accumulation of caspase-14 is associated with keratinocyte differentiation and stratum corneum formation [52]. Our results show that Bax expression was downregulated and Bcl-2, ASK1, and Caspase 14 expression was upregulated in NHKs after treatment with HTSF-exosomes, respectively. Moreover, the proliferation marker Ki-67 was strongly induced. These findings indicate that HTSF-exosomes can regulate proliferation and differentiation of NHKs.

p21 and p27 are important inhibitors of cyclin-dependent kinase, which is associated with keratinocyte differentiation, growth arrest, and terminal differentiation [29]. The downregulation of p21 expression by small interfering RNA transfection reversed the inhibition of the proliferation and migration of carcinoma cells [53]. p21 expression increased in the early stages of differentiated keratinocytes [54] and decreased in spontaneously terminal differentiated keratinocytes, whereas p27 expression remained unchanged [55]. However, compulsively increasing p21 expression inhibited cell growth and decreased expression of differentiation markers loricrin, involucrin, and KRT1, without affecting the cell cycle [55]. These studies suggest that p21 expression could correlate with onset but did not necessarily correlate with the late terminally differentiated phenotype of keratinocytes. Data presented in previous studies demonstrated that the differentiation of murine primary keratinocytes requires p27 induction [56]. A comparative analysis revealed an increased proliferation potential in both p21- and p27-deficient keratinocytes, but to a greater extent in p21-knockout than in p27-knockout keratinocytes. Moreover, the functions of p21 and p27 differ in the expression of differentiation markers in calcium-containing culture. Both p21- and p27-deficient keratinocytes decreased KRT1 expression, while p21-deficient cells alone specifically decreased loricrin and involucrin expression [29]. Therefore, the induction of NHK differentiation by HTSF-exosomes was partially mediated by the upregulation of both p21 and p27 expression.

Several signaling upstream molecules are involved in keratinocyte proliferation and differentiation, such as Notch1, Myc, and PKCη. The Notch signaling controls keratinocyte growth arrest and early differentiation [57]. A detailed in vivo study revealed that the deletion of the Notch1 gene led to dramatically increased epidermal cell proliferation with measurement of Ki-67 and KRT6. Moreover, in vitro, endogenous Notch activity and p21 protein expression increased in differentiating keratinocytes, along with increased extracellular calcium. p21 expression was induced by activated Notch1. Notch1 expression also increased in late terminal differentiating keratinocytes cultured from embryonic mouse skin, compared with those obtained from newborn mouse [31]. c-Myc is a positive regulator of keratinocyte proliferation [58]. Suppression of c-Myc activity inhibited cell proliferation and induced differentiation [59]. Conversely, the induction of c-Myc downregulated p21 expression, which may be one of the mechanisms through which c-Myc promoted cell proliferation [30]. In addition, several studies have established the relationship between PKCη and growth and differentiation of keratinocytes. In PKCη-null keratinocytes, p27 mRNA level was downregulated, and both growth arrest and terminal differentiation were delayed. The re-expression of PKCη or the inhibition of JNK/c-Jun signaling induced the upregulation of p27 mRNA, resulting in cell cycle arrest and terminal differentiation [32]. PKCη could activate Fyn, a Src kinase family member, which is required for normal keratinocyte differentiation. The overexpression of Fyn elevated the expression of p21 and p27, which in turn induced differentiation and growth arrest [60]. Therefore, the results from these studies support our results that HTSF-exosomes increase PKCη and Notch1 to regulate the expression of p21 and p27 and induce NHK differentiation.

EMT is a biophysical molecular process in which epithelial cells lose epithelial cell markers to acquire a mesenchymal cell phenotype, which is followed by an upregulation of mesenchymal cell markers, such as decreased expression of E-cadherin, increased expression of N-cadherin and vimentin, and increased migration [33]. Keratinocyte differentiation has a close relationship with EMT, which is also known as a differentiation program [61]. Previous in vivo studies have demonstrated that vimentin-deficient cells exhibited deprivation of EMT-like keratinocyte activation, poor keratinization, and slow re-epithelialization in wounds that are mediated by transdifferentiation and keratinocyte migration via Slug-EMT signaling in keratinocytes [61]. We found that HTSF-exosomes induce the upregulation of N-cadherin and vimentin expression and migration of NHKs, which is an example of exosomes promoting keratinocyte differentiation.

## 4. Materials and Methods

### 4.1. Primary Cell Culture

The NHKs used in this study were derived from skin biopsy, whereas HTSFs were isolated from burn-injured HTS tissues (size: 10–15 cm × 10–15 cm) obtained during surgical procedures and from normal skin that matched HTS tissue from four patients. The scars ranged from 1–2 years in age. This study was approved by the Hallym University Hangang Sacred Heart Hospital Institutional Review Board (HG 2019-016). Cell culture was performed as previously described [26,39]. Briefly, normal skin and HTS tissues were cut into small pieces, soaked in a Dispase II (1 U/mL; Gibco, Waltham, MA, USA) solution, and maintained at 4 °C overnight. Subsequently, the epidermis was separated from the dermis and digested with 0.125% trypsin (Biowest, Riverside, MO, USA) for 15 min at 37 °C. The separated dermises from normal and HTS tissues were digested with a Collagenase Type IV solution (500 U/mL; Gibco) at 37 °C for 30 min. Both solutions were inactivated with keratinocyte growth medium 2, containing supplements (PromoCell, Heidelberg, Germany), and Dulbecco’s modified Eagle’s medium (DMEM), containing 10% fetal bovine serum (FBS; Biowest) and 1% antibiotic-antimycotic with penicillin, streptomycin, and amphotericin B (Gibco). The solutions were then filtered and centrifuged at 300× *g* for 5 min. Pellets were obtained and resuspended in a complete medium, followed by culture at 37 °C in 5% CO_2_. The NHKs and HTSFs at passage two were used for all experiments.

### 4.2. Exosome Extraction and Treatment

Exosome extraction was performed as described previously [26]. The detailed procedure is as follows. HTSFs at 70% confluence were washed twice with DPBS (Biowest) and then with DMEM containing 10% Exo-depleted FBS (Gibco) for 48 h. Subsequently, the media from the cell culture were collected and centrifuged at 300× *g* for 10 min and at 16,000× *g* for 30 min to remove any remaining cell debris. The supernatants were subjected to centrifugation using Macrosep Advance Centrifugal Devices with a 100 kD omega membrane (Pall Corporation, Port Washington, NY, USA) at 4000× *g* for 1 h. The remaining Exo-containing solutions were collected; exosomes were isolated using an Exo-spin^TM^ midi Purification Kit (Cell Guidance Systems, Cambridge, UK), according to the manufacturer’s instructions. Briefly, the solution was mixed with a 1/2 volume of exosome-spin buffer and incubated overnight at 4 °C. The mixture was then centrifuged at 16,000× *g* for 1 h, carefully aspirated, and the supernatant was discarded. The Exo-containing pellet was resuspended in 1 mL of DPBS, applied to the top of the column (100 μL/column), and centrifuged at 50× *g* for 60 s. PBS (200 µL) was applied to the column and centrifuged at 50× *g* for 60 s. The eluates obtained were considered to contain purified exosomes. The surface markers of exosomes were detected by determining the expression of CD9, CD63, and CD81 [2] using Western blotting. The total protein concentration of exosomes was quantified using a bicinchoninic acid (BCA) protein assay kit (Thermo Scientific, Waltham, MA, USA). The exosomes were stored at –80 °C until use. For the cell proliferation assay and the mRNA and protein expression analysis, NHKs were treated for 24 or 48 h with 100 μg/mL of HTSF-derived exosomes, whereas the control groups were treated with DPBS. For the migration assay, NHKs were treated with the same concentration for 24 or 48 h.

### 4.3. Cell Proliferation Assay

NHK proliferation was assessed using the CellTiter-Glo Luminescent Cell Viability Assay kit (Promega, Madison, WI, USA), as described previously [5]. NHKs were plated in 96-well cell culture plates at a density of 2.0 × 10^4^ cells and cultured for 24 h. They were then treated with exosomes derived from the HTSFs. After 24 or 48 h of cultivation, 100 µL of CellTiter-Glo reagent was added to the medium, mixed well, and incubated for 10 min at room temperature. Luminescence was recorded using a DTX 880 multimode detector (Beckman Coulter, Fullerton, CA, USA). Viability was calculated as follows: viability (%) = (sample luminescence − background luminescence)/(control sample luminescence − background luminescence) × 100.

### 4.4. Quantitative Reverse Transcription-Polymerase Chain Reaction (qRT-PCR)

NHKs were harvested 24 or 48 h after Exo treatment. The total RNA was extracted using the ReliaPrep RNA Miniprep System (Promega), according to the manufacturer’s instructions. The RNA concentration was estimated using a NanoDrop Spectrophotometer (BioTek, Winooski, VT, USA), and 4 µg of RNA was used to generate cDNA with the PrimeScript RT Master Mix (Perfect Real Time) (Takara, Shiga, Japan). Subsequently, 50 ng of cDNA, 0.5 µM primers (Table 1), and a 2× PCR premix (Takara) were used for qPCR on a LightCycler 96 system (Roche, Basel, Switzerland). The reaction conditions were as follows: initial denaturation at 95 °C for 30 s, 40 cycles of amplification at 95 °C for 5 s and 60 °C for 20 s, and extension at 72 °C for 30 s. The mRNA levels of the target genes were normalized to the level of β-actin using the 2^−ΔΔCT^ method [62]. Each qPCR was performed in duplicate with cDNA from four different NHK cultures.

### 4.5. Western Blot Analysis

NHKs were harvested at 24 or 48 h after Exo treatment and lysed in a radioimmunoprecipitation assay buffer containing protease and phosphatase inhibitors (Sigma, St. Louis, MO, USA). The detailed methods have been described previously [63]. The protein concentration was measured using Pierce BCA Protein Assay Kit (Thermo Fisher Scientific). Lysates were mixed with 3× blue loading buffer (Cell Signaling Technology, Danvers, MA, USA) and heated for 3 min at 95 °C. Samples were subjected to gel electrophoresis, electro-transferred onto polyvinylidene difluoride (PVDF) membranes, and blocked with a 5% (*w*/*v*) bovine serum albumin in Tris-buffered saline containing 0.1% Tween-20 (PBST) for 1 h at room temperature. The PVDF membranes were incubated with the primary antibodies (Table 2). Secondary antibodies included horseradish peroxidase (HRP)-conjugated goat anti-rabbit IgG antibody (1:3000; Millipore, Billerica, MA, USA) and HRP-conjugated goat anti-mouse IgG antibody (1:3000; Millipore). Images were obtained using a chemiluminescence imaging system (WSE-6100; ATTO, Tokyo, Japan) and the optical density of the bands was measured using the Image J software (Version 1.53, NIH, Bethesda, MD, USA). Protein expression was normalized to that of β-actin; the ratio of Exo-treated cells to DPBS-treated control cells was calculated (DPBS-treated control cells = 1.0).

### 4.6. Cell Migration Assay

Cell migration was analyzed in a culture insert in a 35-mm µ-dish (ibidi GmbH, Planegg, Munich, Germany), according to the manufacturer’s instructions [64]. To eliminate the impact of cell proliferation during migration, mitomycin C (5 µg/mL; Sigma) was added to the cultures. Images of cell migration were obtained at 24 h (Exo treatment for 48 h) and 48 h (Exo treatment for 72 h) after migration under a light microscope (IX 70, Olympus, Tokyo, Japan); the number of cells that had migrated into the gap (cell-free wound) was analyzed using the Image J software v 1.53 (NIH, Bethesda, MD, USA). Exo-treated cells were compared with untreated NHKs, which served as a control, for which migration was set to 100%. Each analysis was performed in triplicate.

### 4.7. Statistical Analyses

All results are presented as the mean ± SD. The Mann-Whitney U test was used for making comparisons between the PBS-treated control group and the Exo-treated group. Statistical analyses were performed using PASW statistics 24 (SPSS Inc., Chicago, IL, USA). *p*-value < 0.05 was considered statistically significant.

## 5. Conclusions

We observed that exosomes derived from HTSFs dominantly promote the proliferation, differentiation, and activation of human normal keratinocytes, compared with exosomes derived from NFs. Therefore, our results indicate that HTSF-exosomes contribute to the epidermal development of HTS, where it is hyper-proliferated and hyperactivated, and has an alternative differentiation program.

## Figures and Tables

**Table 1:** Sequences of primers used in real-time PCR.

Gene	Accession No	Forward (5′ → 3′)	Reverse (5′ → 3′)
*KRT1*	NM_006121.4	TGGATGGTGCTTATATGAC	GACAACTCTGCTTGGTAG
*KRT6A*	NM_005554.4	TGAAGAAGGATGTGGATG	ATCATACAAGGCTCTCAG
*KRT10*	NM_000421.5	GATTCTCAACCTAACAACTG	GCTACCTCATTCTCATACT
*KRT14*	NM_000526	GCTGAGATCAAAGACTACA	AGAAGGACATTGGCATTG
*KRT16*	NM_005557.4	CCTACTTCAAGACCATCG	CCTGGCATTGTCAATCTG
*KRT17*	NM_000422.3	ATCCTGCTGGATGTGAAGACGC	TCCACAATGGTACGCACCTGAC
*MAP3K5*	NM_005923.4	CGTGAGCACGCTCAGTTCTA	TTCCGAACCAATTCTTCCAG
*NOTCH1*	NM_017617.5	AGCCTCAACGGGTACAAG	TTGACACAAGGGTTGGATTC
*CASP14*	NM_012114.3	CCTGTTGTCACCTTGCTAT	GTCCTTGCCTCTGTCTTAC
*BAX*	NM_001291428.2	CCTTTTGCTTCAGGGTTTCA	CCATGTTACTGTCCAGTTCG
*BCL2*	NM_000633.3	TGCGGCCTCTGTTTGATTT	AGGCATGTTGACTTCACTTGT
*CDKN1A*	NM_078467.3	TAGGCGGTTGAATGAGAG	AAAGGAGAACACGGGATG
*CDKN1B*	NM_004064.5	GCAGGAATAAGGAAGCGA	GGGAACCGTCTGAAACAT
*PRKCH*	NM_006255	CGCCATCTTGAGACATCCTT	TTCTCGGGATTTGATTCTGG
*VIM*	NM_003380.5	AAAGCGTGGCTGCCAAGAA	ACCTGTCTCCGGTACTCGTTTGA
*CDH1*	NM_004360.5	GCAGACCTTCCTCCCAATAC	TGGGTCGTTGTACTGAATGG
*CDH2*	NM_001792.5	CCACCTTA AAATCTGCAGGC	CCATGTTACTGTCCAGTTCG
*MYC*	NM_002467.6	CCTGGTGCTCCATGAGGAGAC	CAGACTCTGACCTTTTGCCAGG
*GAPDH*	NM_ 002046.7	CATGAGAAGTATGACAACAGCCT	AGTCCTTCCACGATACCAAAGT

**Table 2:** Primary antibodies used in Western blot analysis.

Target	Host	Dilution	Company (Cat. No.)
CD 9	Mouse	1:250	Invitrogen (10626D), Waltham, MA USA
CD 63	Rabbit	1:1000	System Biosciences (EXOAB-CD63A-1), Palo Alto, CA, USA
CD 81	Mouse	1:250	Invitrogen (10630D), Waltham, MA, USA
Ki-67	Rabbit	1:1000	Abcam (ab16667), Cambridge, UK
Keratin 1	Rabbit	1:1000	Abcam (ab93652), Cambridge, UK
Keratin 6	Mouse	1:2000	Abcam (ab18586), Cambridge, UK
Keratin 10	Rabbit	1:500	Santa Cruz Technology (sc-23877), Dallas, TX, USA
Keratin 14	Rabbit	1:2000	Abcam (ab181595), Cambridge, UK
Keratin 16	Rabbit	1:2000	Abcam (ab76416), Cambridge, UK
Keratin 17	Rabbit	1:2000	Abcam (ab8068), Cambridge, UK
ASK 1	Rabbit	1:1000	Abcam (ab45178), Cambridge, UK
Notch 1	Rabbit	1:1000	Abcam (ab52627), Cambridge, UK
Caspase 14	Rabbit	1:1000	Abcam (ab174847), Cambridge, UK
Bax	Rabbit	1:1000	Abcam (ab199677), Cambridge, UK
Bcl2	Rabbit	1:1000	Abcam (ab196495), Cambridge, UK
P21	Rabbit	1:1000	Abcam (ab109199), Cambridge, UK
P27	Rabbit	1:1000	Abcam (ab32034), Cambridge, UK
PKCη	Rabbit	1:1000	GenScript (A00906), Piscataway, NJ, USA
Vimentin	Rabbit	1:2000	Abcam (ab92547), Cambridge, UK
E-cadherin	Rabbit	1:1000	Cell Signaling Technology (3195S), Danvers, MA, USA
N-cadherin	Mouse	1:1000	Thermo Fisher Scientific (33-3900), Waltham, MA, USA
MYC	Rabbit	1:1000	Cell Signaling Technology (9402), Danvers, MA, USA
β-actin	Rabbit	1:2000	Cell Signaling Technology (4967S), Danvers, MA, USA
β-actin	Mouse	1:1000	Santa Cruz Technology (sc-47778), Dallas, TX, USA

## Supplementary Materials

The following supporting information can be downloaded at: https://www.mdpi.com/article/10.3390/ijms24076132/s1.
