# Supplementary material for: Effect of Hypertrophic Scar Fibroblast-Derived Exosomes on Keratinocytes of Normal Human Skin
Source: Int J Mol Sci. 2023 Mar 24;24(7):6132. doi: 10.3390/ijms24076132 (PMC10094451; doi:10.3390/ijms24076132)
Supplement: Supplementary file 1 [file ijms-24-06132-s001.zip › ijms-1983258-supplementary.pdf]

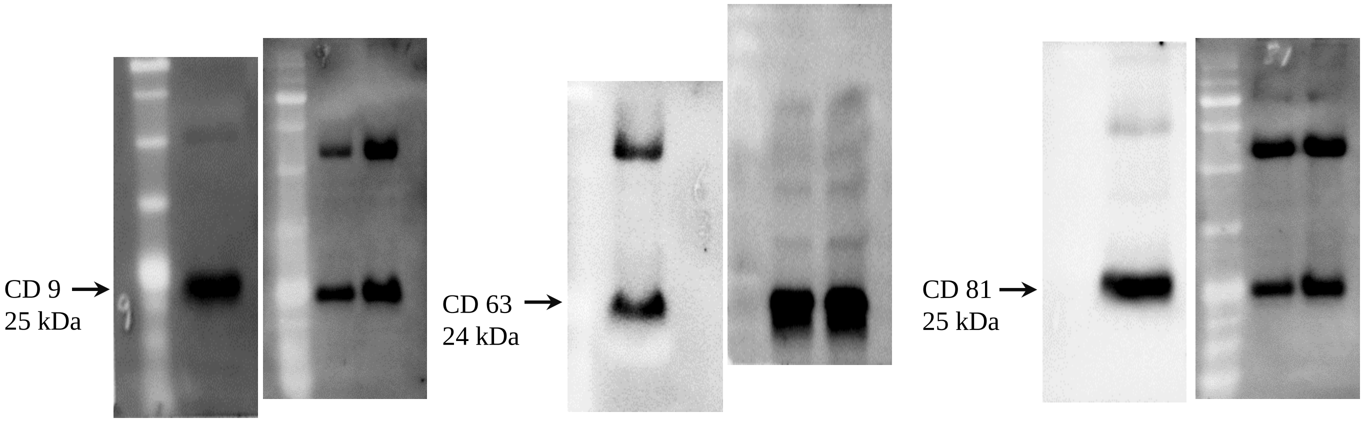

Figure S1
The original images of western blotting for CD9, CD63, and CD81.

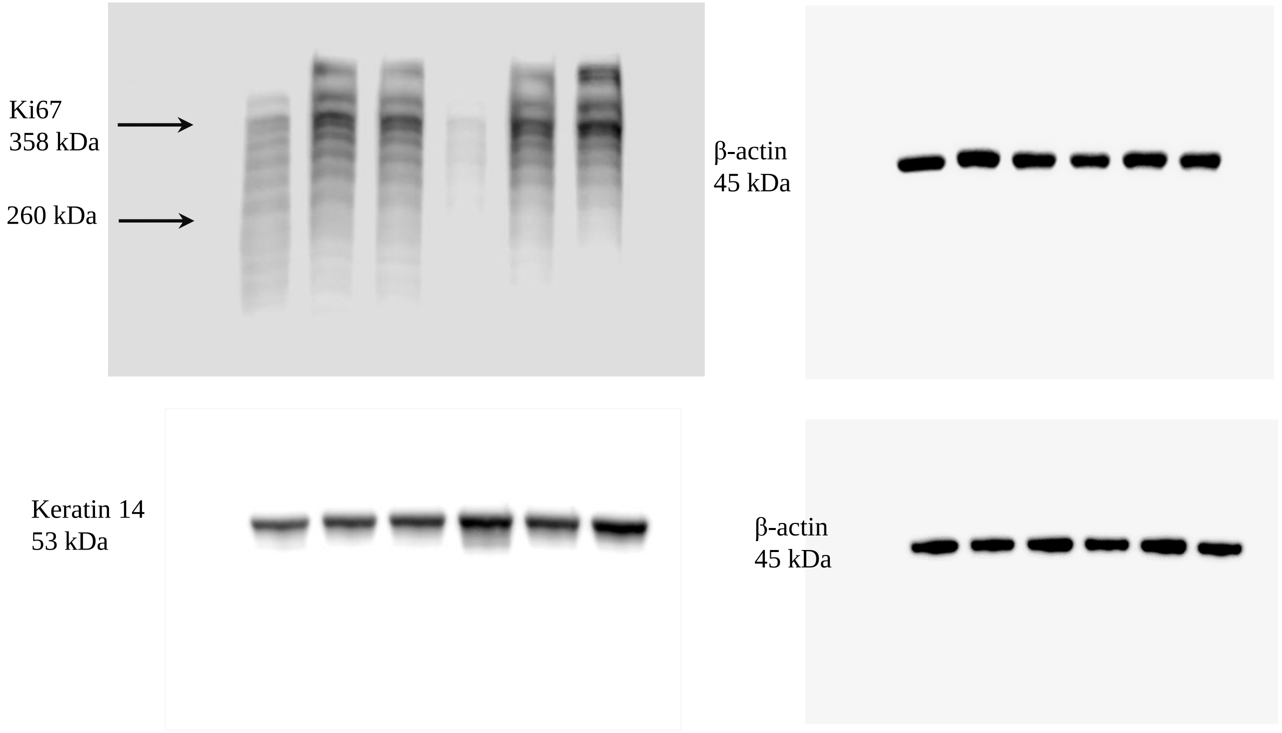

Figure S2
The original images of western blotting for Ki67, and keratin 14, and β-actin.

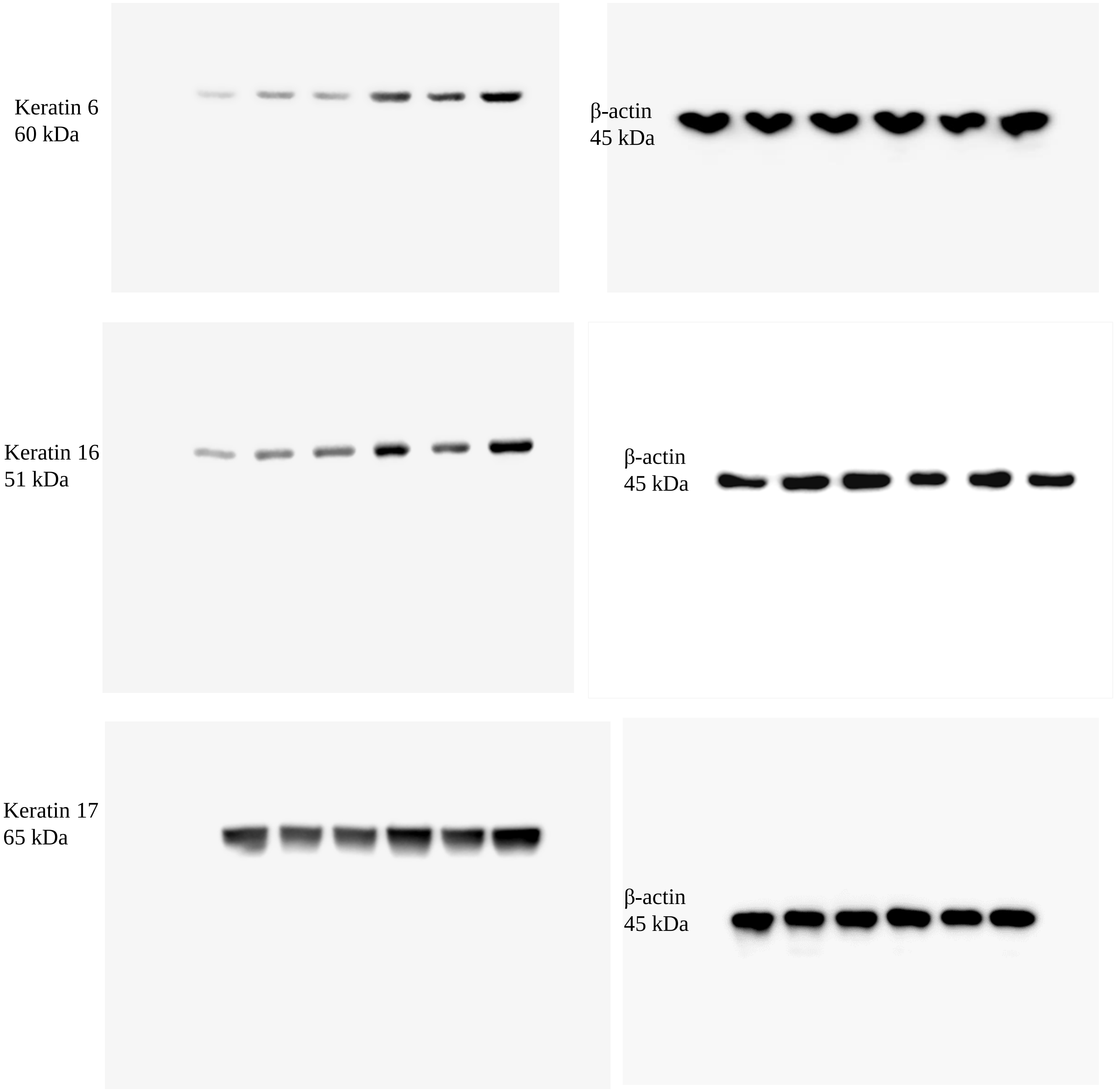

Figure S3

The original images of western blotting for keratin 6, keratin 16, keratin 17, and β-actin.

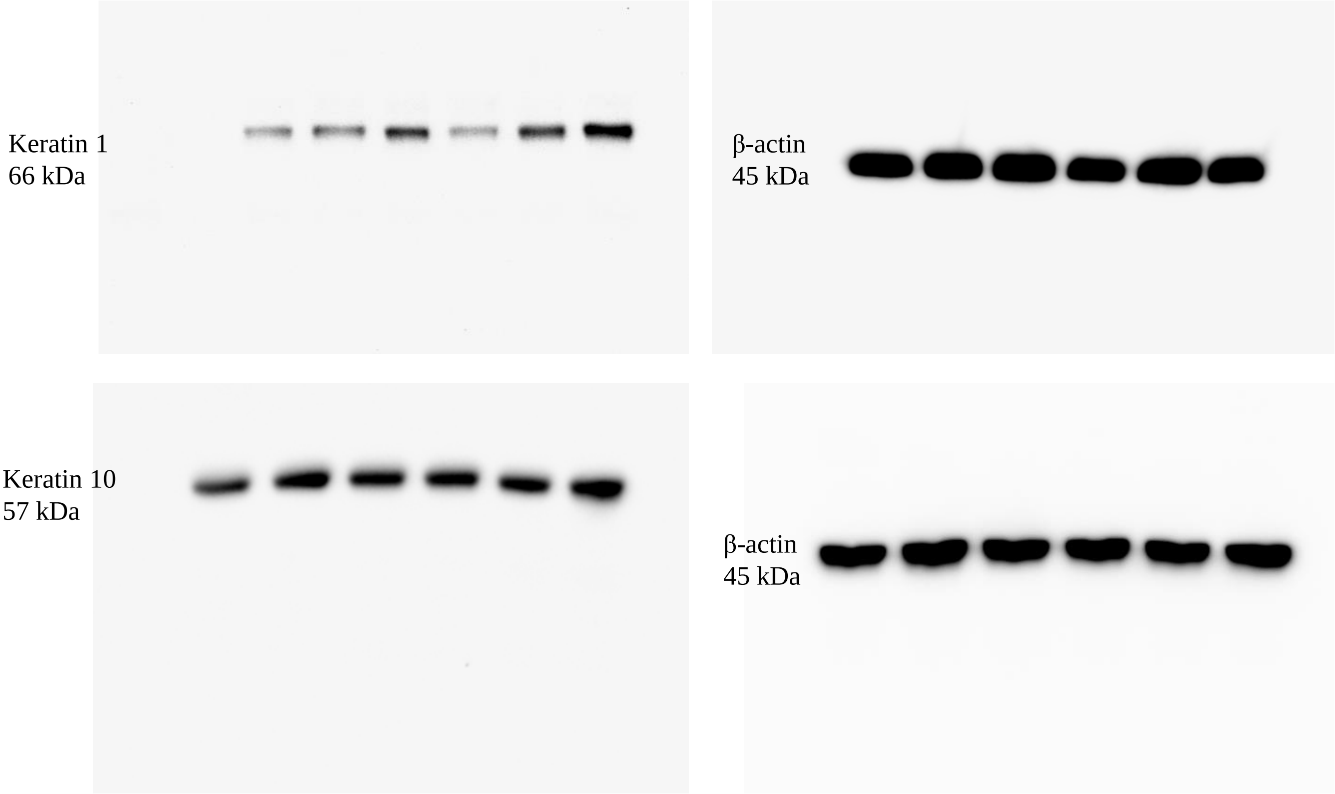

Figure S4
The original images of western blotting for keratin 1, keratin 10, and β-actin.

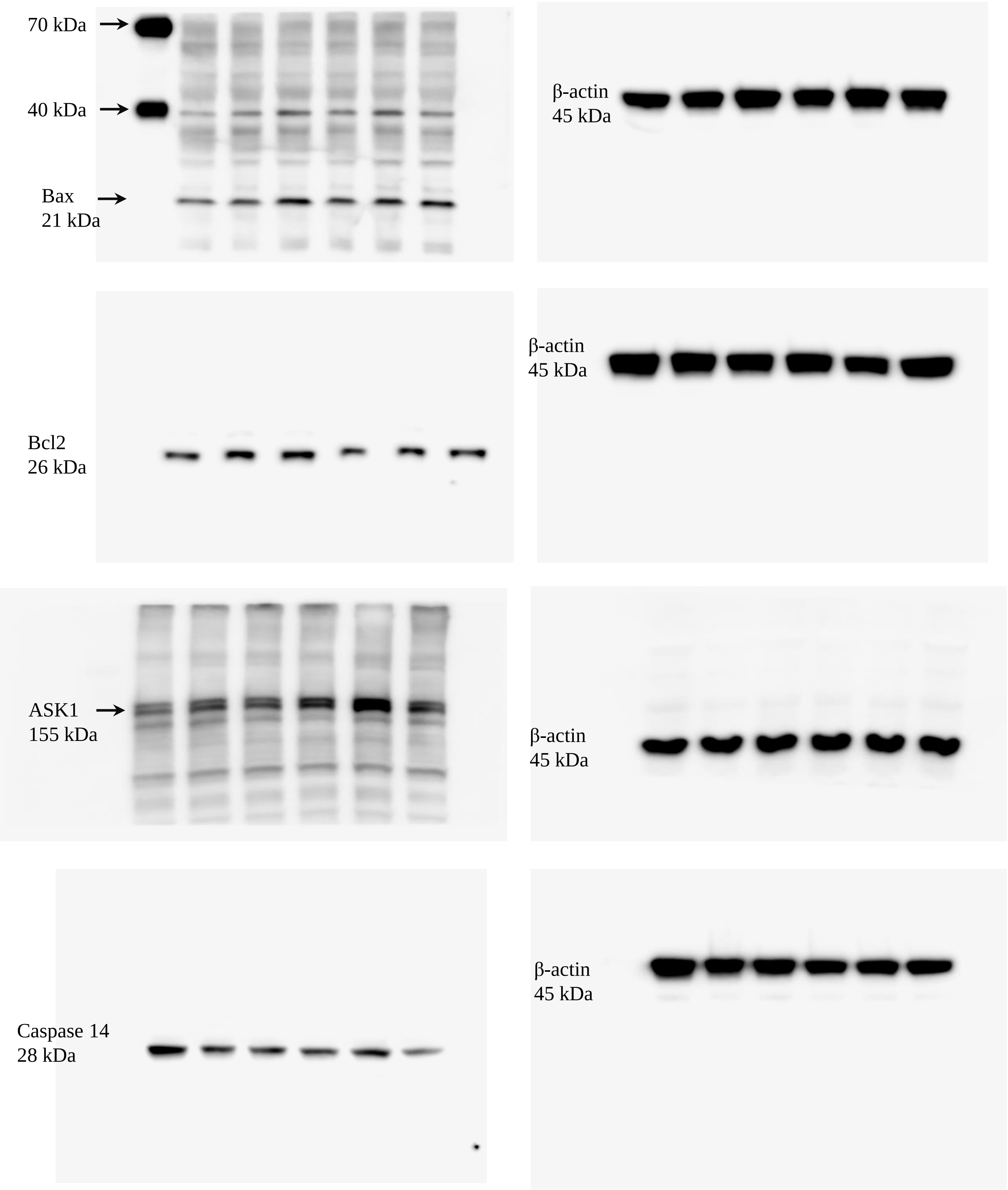

Figure S5
The original images of western blotting for bax, bcl2, caspase 14, and β-actin.

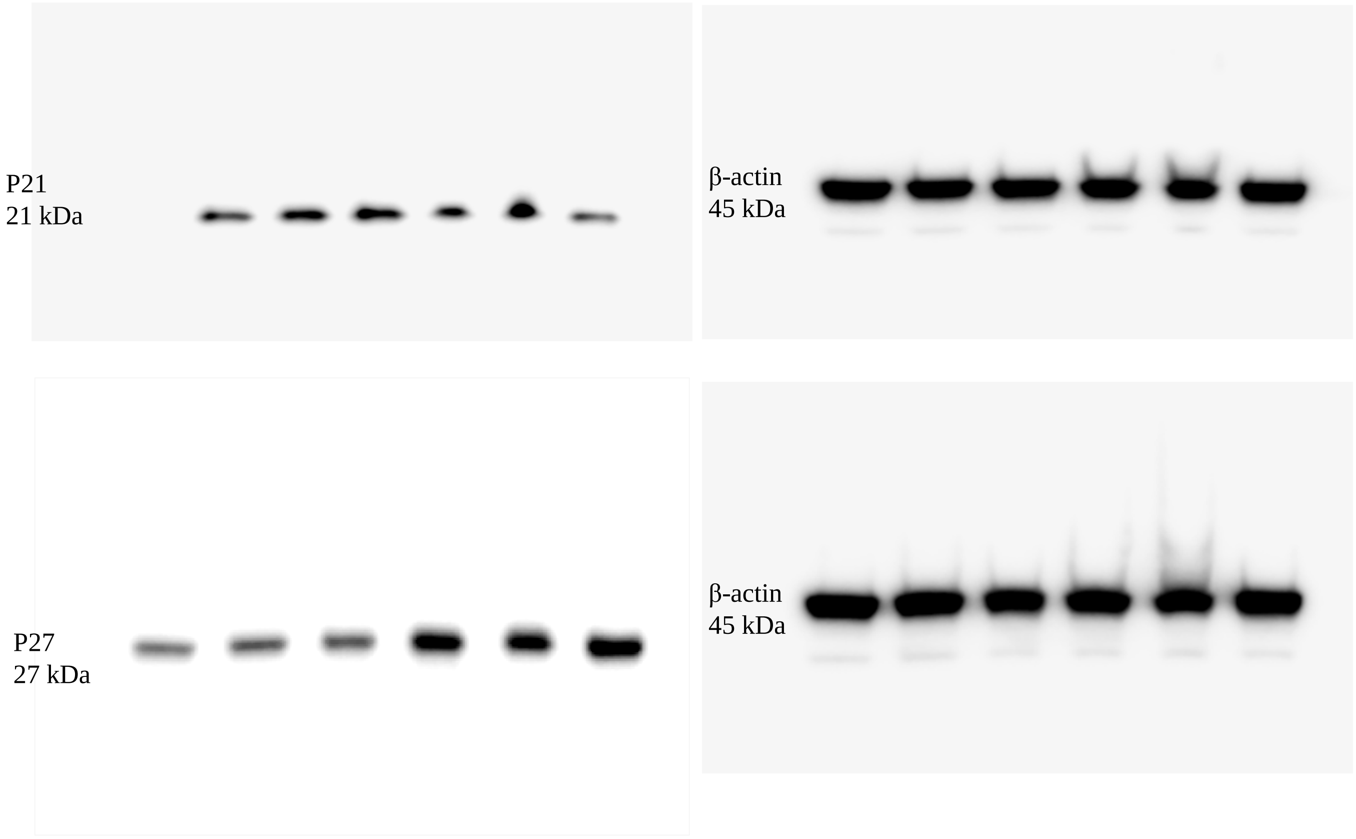

Figure S6
The original images of western blotting for P21, P27, and β-actin.

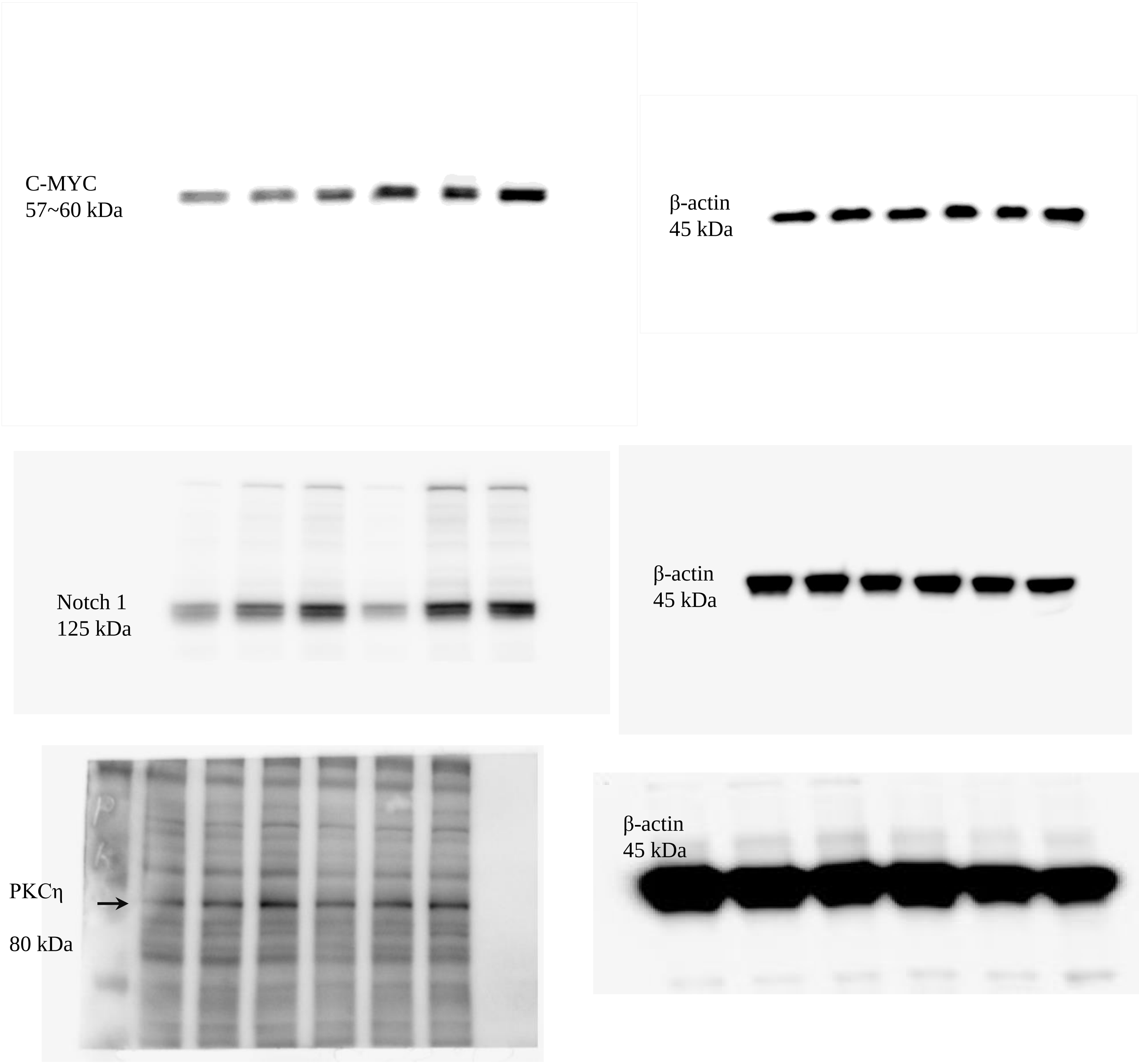

Figure S7
The original images of western blotting for C-MYC, Notch 1, PKCη, and β-actin.

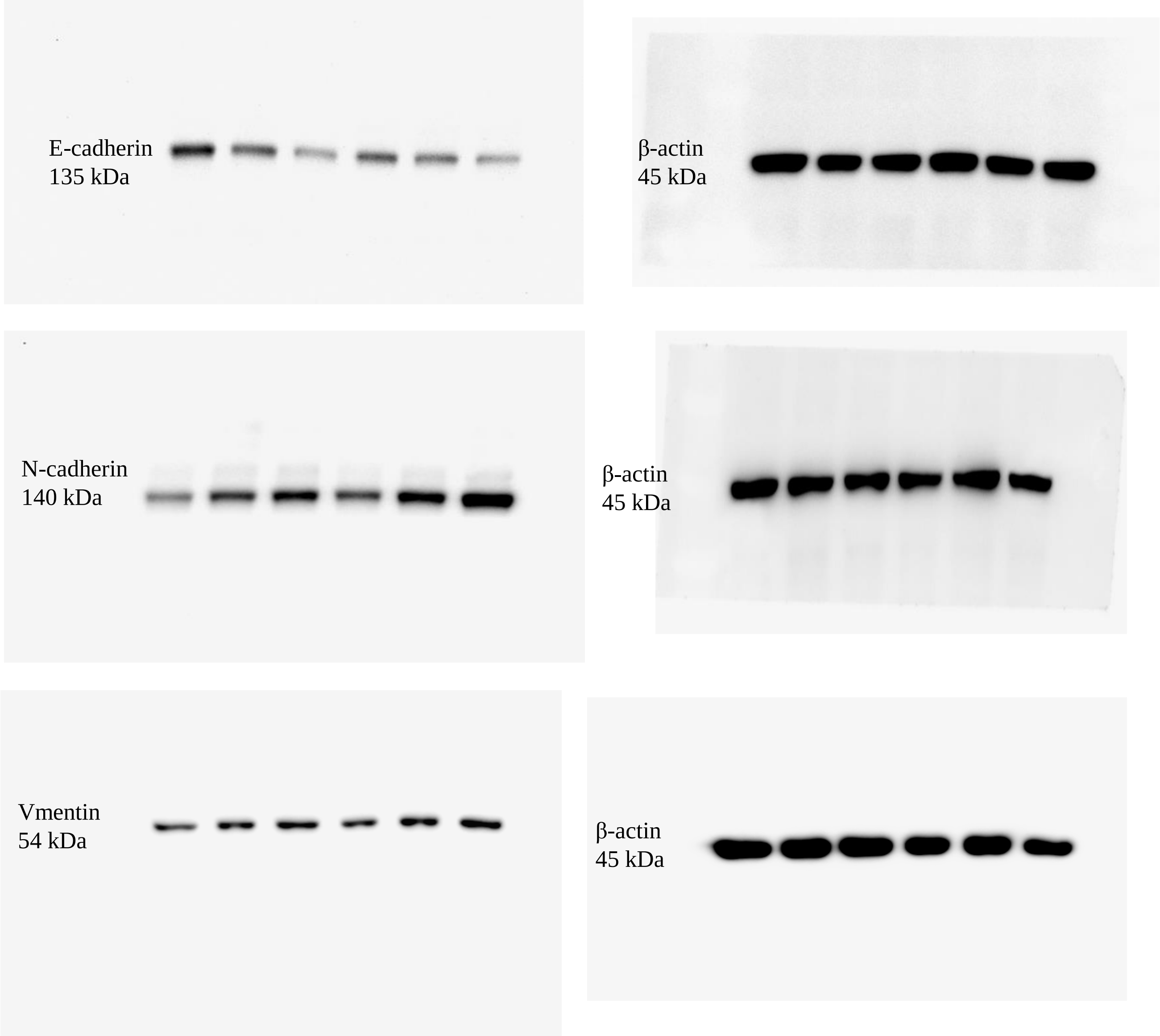

Figure S8
The original images of western blotting for E-cadherin, N-cadherin, vimentin, and β-actin.
